# Production and Use of Recombinant Profilins Amb a 8, Art v 4, Bet v 2, and Phl p 12 for Allergenic Sensitization Studies

**DOI:** 10.3390/molecules25020369

**Published:** 2020-01-16

**Authors:** Beata Cudowska, A. Brenda Kapingidza, Magdalena Pawłowicz, Agnieszka Pampuch, Noah Hyduke, Swanandi Pote, Caleb R. Schlachter, Dariusz M. Lebensztejn, Maksymilian Chruszcz, Krzysztof Kowal

**Affiliations:** 1 Department of Pediatrics, Gastroenterology, Hepatology, Nutrition and Allergology, Medical University of Bialystok, 15-276 Bialystok, Poland; beata.cudowska@umb.edu.pl (B.C.); magdalena_pawlowicz@o2.pl (M.P.); lebensztejn@hoga.pl (D.M.L.); 2 Department of Chemistry and Biochemistry, University of South Carolina, Columbia, SC 29208, USA; anyway@email.sc.edu (A.B.K.); nhyduke@email.sc.edu (N.H.); swanandi@gmail.com (S.P.); schlachc@gmail.com (C.R.S.); 3 Department of Allergology and Internal Medicine, Medical University of Bialystok, 15-276 Bialystok, Poland; agapam@o2.pl; 4 Department of Experimental Allergology and Immunology, Medical University of Bialystok, 15-276 Bialystok, Poland

**Keywords:** recombinant allergen, IgE, pollen-food allergy, profilin

## Abstract

Four recombinant (r) allergens (rAmb a 8.0101, rArt v 4.0101, rBet v 2.0101, and rPhl p 12.0101) were successfully produced and used for sensitization studies. The allergens belong to the profilin family which is one of the most numerous allergen families. These four proteins represent allergens originating from pollen of weeds (rAmb a 8.0101 and rArt v 4.0101), tree (rBet v 2.0101) and grass (rPhl p 12.0101). The recombinant allergens were characterized using various biochemical and biophysical methods and tested for their ability to bind patient-derived antibodies. One hundred patients aged 2 to 50 years sensitized to pollen and plant-derived food allergens (IgE > 0.35 kU/L) were included. Sensitization to individual allergen sources and components of birch and timothy pollens was evaluated using multiparameter immunoblots. The presence of IgE to pollen-derived recombinant profilins rAmb a 8.0101, rArt v 4.0101, rBet v 2.0101, and rPhl p 12.0101 in serum was evaluated using ELISA method. The presence of IgE against pollen profilins was detected in 20 out of 100 studied patients. High correlation was seen between IgE ELISA results with individual pollen profilins. In summary, it was shown that the recombinant versions of the four allergenic profilins can be used for sensitization studies and for component-resolved allergy diagnostics.

## 1. Introduction

**Figure 1:**
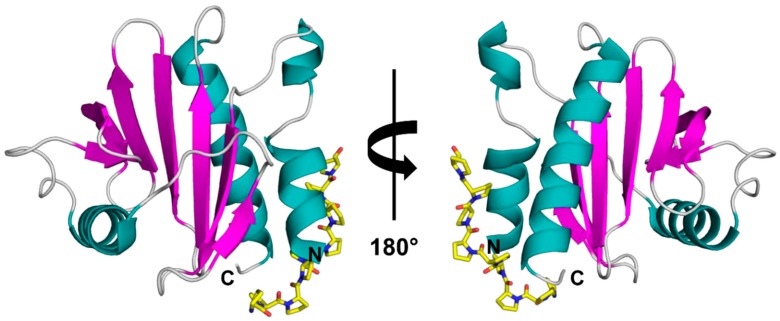
Crystal structure of rArt v 4.0101 (PDB code: 5EM0) with modelled poly(L-Pro). The structure is shown in two different orientations with secondary structure elements colored (α-helices—teal, β-strands—purple, loops—grey). The poly(L-Pro) is shown in yellow stick representation.

Profilins are ubiquitous, small proteins ranging between 12–16 kDa that are expressed in all eukaryotic cells and certain viruses, with the exception of some protists [1,2]. Profilins play a role in regulating various cellular processes like membrane trafficking, actin cytoskeletal dynamics, and they bind to proline-rich regions of proteins (Figure 1) [3]. The profilin family shares highly conserved amino acid sequences, even among distantly related sources [4]. This also holds true for profilins derived from different plants that are highly similar to each other, sharing approximately 80% amino acid identity [4]. This renders them as panallergens, but minor allergens widespread in pollens and foods, and responsible for immunoglobulin E (IgE) cross-reactivity [5]. Moreover, structural and immunological analyses of profilins indicate that profilins present in pollens and plant-derived foods are highly cross-reactive [4]. These proteins usually act as respiratory allergens producing mild allergic symptoms [6,7].

Under certain circumstances, however, they may be responsible for food-derived allergy symptoms including systemic anaphylaxis [6,7,8]. A broad range of plant-derived food products contain profilins, including fruits, legumes, nuts, and vegetables [1,4]. It is commonly accepted, however, that profilin-dependent allergic reactions to food result from cross-reactivity between pollen and food profilins where the former are true sensitizers [6,7]. Although some profilins can elicit adverse immune responses like anaphylaxis, the infrequent systemic reactions observed in patients after oral exposure to profilins is due to their low stability and high susceptibility to denaturation by proteases and the acidic environment in the digestive tract [9,10].

**Figure 2:**
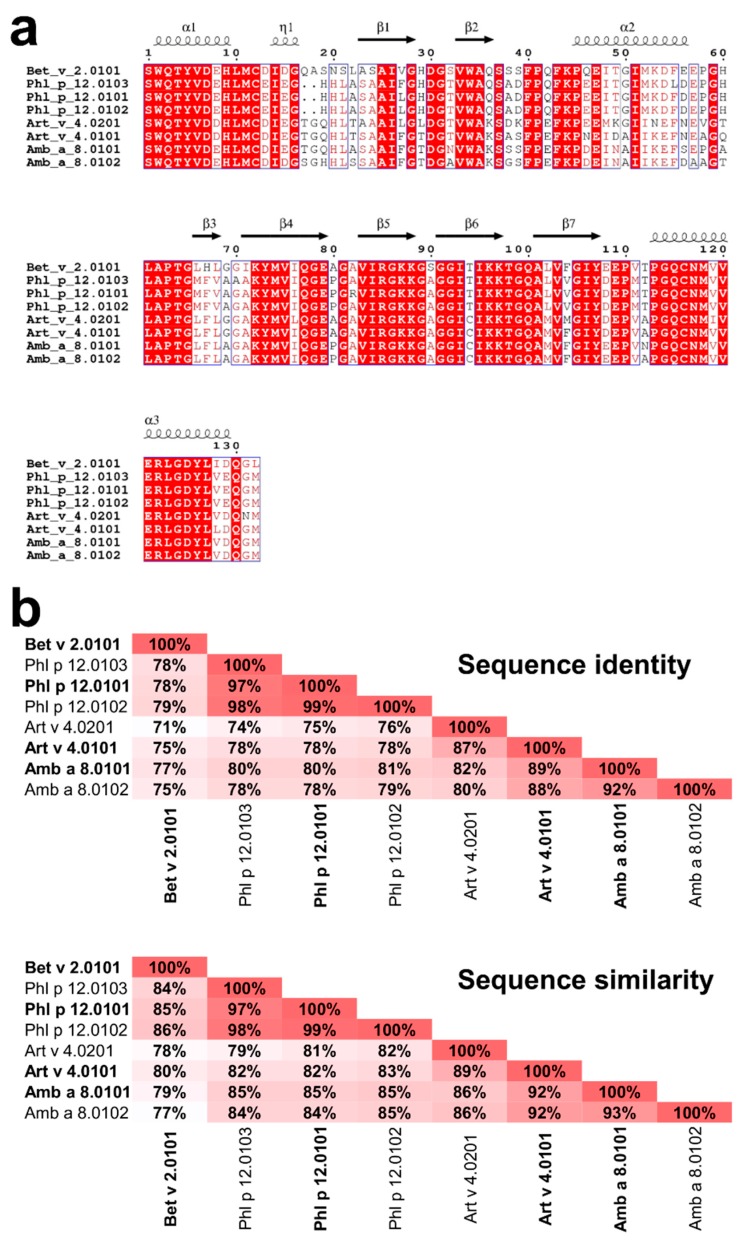
Comparison of Amb a 8, Art v 4, Bet v 2, and Phl p 12 sequences. All isoallergens reported by the World Health Organization (WHO) and International Union of Immunological Societies (IUIS) Allergen Nomenclature Sub-committee (allergen.org) are shown. (**a**) Sequence alignment generated using Clustal Omega [17] and ESPript [18]. (**b**) Sequence identities and similarities between the profilins as calculated by SIAS (http://imed.med.ucm.es/Tools/sias.html) using the default parameters.

Profilins represent one of the most numerous allergen families that are associated with many clinical syndromes, including pollen-food syndrome discussed above [11]. Of particular interest are profilins emanating from weed pollens, plants, and grass that play a major role in eliciting seasonal allergies [12]. In this research, four profilins, two from weeds (Amb a 8 and Art v 4), one from trees (Bet v 2), and one from grass (Phl p 12), were studied (Figure 2). Amb a 8 originates from short ragweed (*Ambrosia artemisiifolia*), and Art v 4 originates from mugwort (*Artemisia vulgaris*). Although the Asteraceae family is comprised of thousands of plant species, Ambrosia (ragweed) and Artemisia (mugwort) are among the most important sources of profilins [12]. Mugwort is a member of the Asteraceae family found in the northern hemisphere, the Mediterranean basin, and parts of Asia [13]. In late summer and autumn, mugwort pollen is primarily responsible for allergic reactions in patients with mugwort allergy [2]. Specifically in Europe, Art v 4 has a sensitizing prevalence of 34–36% [12]. In late summer and early fall, ragweed elicits type 1 allergic immune responses in more than 15 million patients in just the USA and Canada [12]. Amb a 8 has high amino acid sequence identity (89%) and similarity (92%) when compared to Art v 4 (Figure 2), which implicates a very high likelihood of cross reactivity according to the A-RISC index [4].

Bet v 2 (birch pollen allergen) was the first profilin shown to be an allergen [14]. It is a 15 kDa protein originating from *Betula pendula* also known as the European white birch. Of the 242 birch pollen-sensitized patients from six European countries, 22% recognized Bet v 2 by IgE antibody production [14]. Like most profilins, this qualifies Bet v 2 as a minor allergen. Although a minor allergen, Bet v 2 plays a major role in IgE cross-reactivity between plants and food [15]. On the other hand, Phl p 12 originates from *Phleum pratense*, Timothy grass, a perennial grass native to most of Europe and found mainly in the Pacific Northwest, the Northeast, and Midwest of the United States. Due to this ubiquitous nature of Timothy grass, it is a potent source of allergens for many patients sensitized to the grass. Despite Phl p 12′s characterization as a minor allergen, it displays comparable T cell response prevalence and strength as Phl p 1, a major allergen from *P. pratense* [16]. In addition, it is released from Timothy pollen in the same timeframe as the other Timothy grass allergens and in similar amounts as the major allergen, Phl p 1 [16].

The main scope of this research was to produce the recombinant versions of Amb a 8.0101, Art v 4.0101, Bet v 2.0101, and Phl p 12.0101, characterize the molecular and physical properties of these profilins, and finally test their sensitizing patterns in children and adult patients from North-East Poland. The region is abundant in birch, grass, and mugwort, but no ragweed is to be found. Consequently, studying the molecular and immunological characteristics of Amb a 8, Art v 4, Bet v 2, and Phl p 12 will clarify the clinical relevance of these important profilins in allergy sensitization and their possible contribution to pollen-food syndrome.

## 2. Results

### 2.1. Protein Production and Thermal Stability

The yield of recombinant Phl p 12.0101 production was approximately 70 mg of protein per 1 L of *Escherichia coli* culture. Protein recovery after cleavage of the purification tag by TEV protease was observed to be about 70%. SDS-PAGE confirmed the purity of the protein to be >98%. The protein was purified in monomeric form and mass spectrometry (results not shown) confirmed the expected molecular weight for this quaternary structure. The final yield of the rPhl p 12.0101 production is approximately two-fold higher when compared with the yield of production of rAmb a 8.0101, rArt v 4.0101, rBet v 2.0101, and rCuc m 2.0101 [2,10].

**Figure 3:**
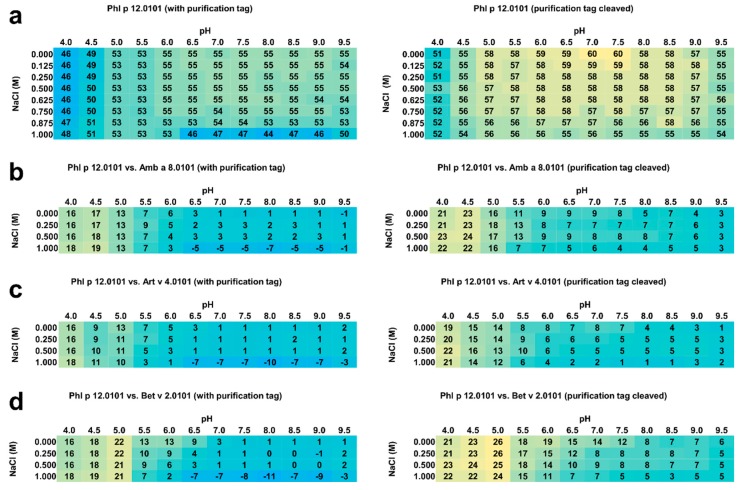
(**a**) Average melting temperatures (T_m_ in °C) for rPhl p 12.0101 with and without purification tag. Yellow and blue represent high and low melting temperatures, respectively. (**b**) Melting temperature (T_m_ in °C) differences between rPhl p 12.0101 and rAmb a 8.0101. (**c**) Melting temperature differences between rPhl p 12.0101 and rArt v 4.0101. (**d**) Melting temperature differences between rPhl p 12.0101 and rBet v 2.0101. Yellow and blue represent high and low differences in the melting temperatures, respectively. The standard deviation for the presented values was less than 1 °C for all experiments.

For the thermal stability of rPhl p 12.0101, the DSF results performed show that the protein without purification tag was more stable with T_m_ difference as high as 6 °C than with the tag intact (Figure 3a). Both versions of the protein, however, are most stable between pH 5.0 to 8.5 and least stable between pH 4.0 to 4.5. Salt concentration does not have a major impact on rPhl p 12.0101 thermal stability, and the only exemption is seen at 1 M NaCl for the protein with the purification tag.

Generally, in comparison with rAmb a 8.0101, rArt v 4.0101, and rBet v 2.0101, rPhl p 12.0101 without purification tag is more stable than all the other three profilins (Figure 3b–d). However, the timothy grass profilin is significantly more stable in solutions with lower pH values both with and without the purification tag. For example, when rPhl p 12.0101 is compared to the other three profilins, the protein is clearly more stable in the 4.0 to 6.0 pH range, and the differences are especially pronounced in solutions with pH between 4.0 and 5.5. In these conditions, rPhl p 12.0101 has T_m_ 7–26 °C higher in comparison with rAmb a 8.0101, rArt v 4.0101, and rBet v 2.0101 (Figure 3b–d). The biggest differences are observed for solutions with pH range 4.0 to 5.0. On the other hand, rPhl p 12.0101 with the purification tag is nearly as stable as the other three profilins from pH 7.0 to 9.5. Interestingly, at 1 M salt concentration, rPhl p 12.0101 with purification tag is significantly less stable than rAmb a 8.0101, rArt v 4.0101, and rBet v 2.0101 with T_m_ difference as high as 11 °C (Figure 3d).

### 2.2. CD Spectroscopy

The secondary structure for Phl p 12 was determined by CAPITO software based on CD spectroscopy data. The secondary structure for the protein was estimated as follows (according to similarity hits based on 25 nearest neighbors): α-helices 9–16%, β-strands 32–48%, and random coils (irregular) 42–58% [19]. These results are very similar to the secondary structure calculated using the crystal structure of another profilin reported previously, Cuc m 2.0101 [10], and in addition, the CD spectra for rPhl p 12.0101 is very similar to that recorded for Cuc m 2. These data strongly suggest that the rPhl p 12.0101 is properly folded.

### 2.3. Profilin Sensitization

**Figure 4:**
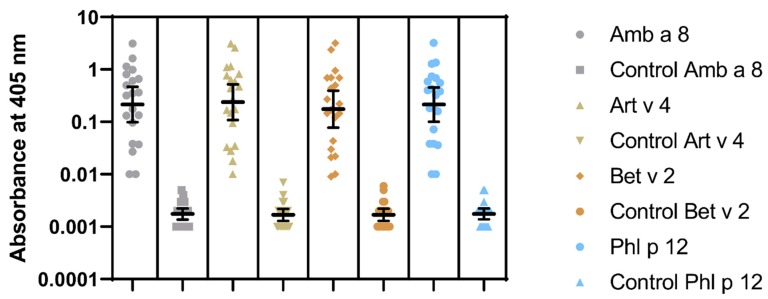
IgE ELISA results to recombinant Amb a 8.0101, Art v 4.0101, Bet v 2.0101, and Phl p 12.0101. Geometric means with 95% CI are marked.

**Figure 5:**
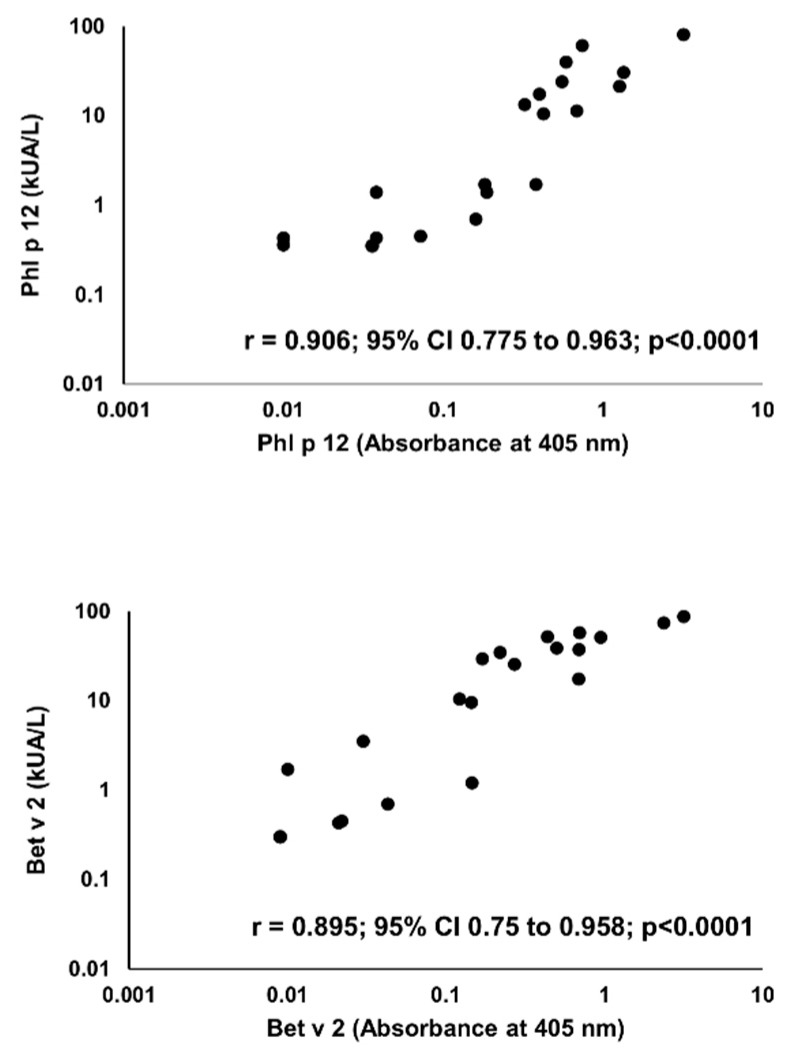
Correlation between IgE ELISA (X-axis) and multiparameter immunoblot (Y-axis) results for Phl p 12 (**upper panel**) and Bet v 2 (**lower panel**).

Sensitization to profilins was demonstrated in 20 (P+) out of 100 pollen and/or food-allergen-sensitized subjects, as determined by the commercial component multiblot. There were 9 P+ among 50 studied children (18%) and 11 P+ among 50 studied adults (22%). Similar results were obtained in the IgE ELISA. The mean absorbances of IgE ELISA with individual pollen profilins rAmb a 8.0101, rArt v 4.0101, rBet v 2.0101, and rPhl p 12.0101 in P+ were significantly greater than those in P- (Figure 4). The results of IgE ELISA with rBet v 2.0101 and rPhl p 12.0101 significantly correlated with the corresponding results of multiparameter immunoblots (r = 0.895; 95% CI 0.75 to 0.958, *p* < 0.0001 and r = 0.906; 95% CI 0.775 to 0.963, *p* < 0.0001, respectively) (Figure 5).

**Figure 6:**
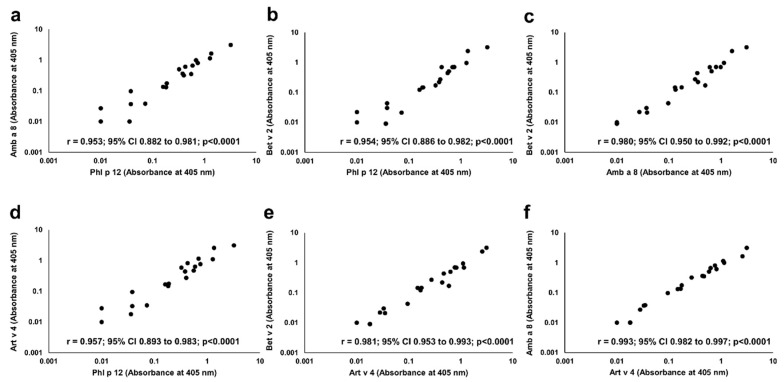
Correlation between IgE binding to individual recombinant profilins. Pairwise comparison of IgE ELISA results performed with Amb a 8 (**a**,**c**,**f**), Art v 4 (**d**,**e**,**f**), Bet v 2 (b, c, e) and Phl p 12 (**a**,**b**,**d**) is depicted on individual charts.

In each of P+ patients, IgE reactivity to all four studied profilins (rAmb a 8.0101, rArt v 4.0101, rBet v 2.0101, and rPhl p 12.0101) was found. Significant correlations were seen between the IgE ELISA results with each individual profilin (Figure 6).

**Figure 7:**
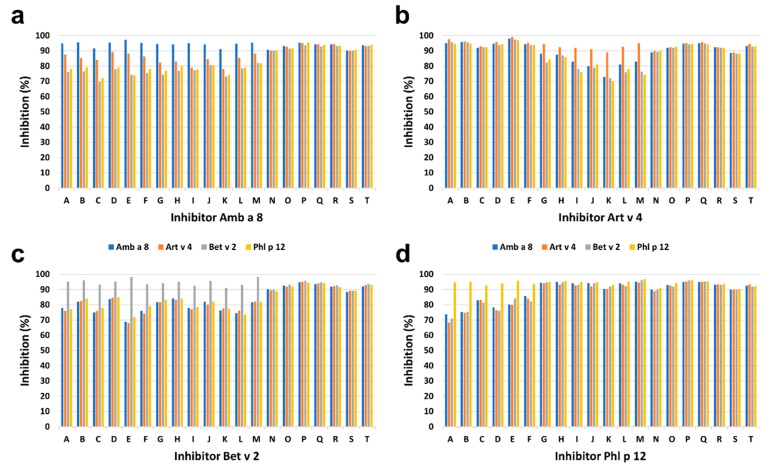
Results of ELISA inhibition assays, in which Amb a 8.0101 (**a**), Art v 4.0101 (**b**), Bet v 2.0101 (**c**), or Phl p 12.0101 (**d**) were used in increasing concentrations. Maximum inhibition of IgE binding by different profilins in individual patients is shown. Letters represent individual patients. Patients A–F: dominant IgE binding to Art v 4.0101; G–M: dominant IgE binding to Phl p 12.0101; N–T: no dominant IgE binding.

The intensity of IgE binding to individual recombinant profilins was different among P+ patients. The greatest intensity of IgE binding to Phl p 12.0101 was demonstrated in 7 of 20 (35%) P+ patients including four children and three adults, and to Art v 4.0101 in 6 of 20 (30%) P+ patients including three children and three adults. The results were supported by inhibition ELISA (Figure 7). Complete inhibition of IgE binding exclusively by Phl p 12.0101 and Art v 4.0101 was demonstrated in 7 of 20 (35%) and 6 of 20 (30%) P+ patients, respectively (Figure 7). However, in seven patients (five adults and two children), no clear dominance of IgE reactivity to a single profilin could be demonstrated.

## 3. Discussion

Profilins are panallergens that are often associated with co-sensitization and various syndromes which arise due to the profilins’ IgE cross-reactivity. Due to no reports of posttranslational modifications of allergenic profilins, these group of proteins can be efficiently produced in *E. coli* and used in a recombinant form for component-resolved allergy diagnostics. In addition, the recombinant version of these proteins can be used to determine sensitization patterns characteristic to various regions [20,21,22]. The use of recombinant profilins provides an opportunity for standardization of tests and eliminates problems associated with the low content of these allergens in some extracts [23,24]. At the same time, one has to take the relatively low stability of these proteins into account when storing the recombinant profilins or using them in solutions that have very low or very high pH values [10]. Here, we have demonstrated that rPhl p 12.0101 is significantly more stable than other pollen profilins and can be used in a relatively broad range of pH values. Therefore, this allergen seems to a be viable candidate for application in component-resolved allergy diagnostics.

The prevalence of sensitization to profilins in our population was similar to that reported previously in other populations of patients suffering from pollen allergy [22,25,26,27]. Sensitization to all studied profilins detected in each P+ patient reflected high structural similarities and immune cross-reactivity among plant-derived profilins (Figure 2) [2]. However, the prevalence of IgE sensitization to different cross-reactive allergens is different in different populations and is influenced by several factors such as geographic factors, patient age, and sensitization patterns [22,25,26,27,28]. Interestingly, in one population of vegetable food-allergic patients in Italy, sensitization to profilins was as low as 11.7% [25]. On the other hand, others reported frequency of sensitization to profilins in children with pollen-food syndrome greater than 30% which was significantly greater than that in rhinoconjunctivitis without allergy to food [16]. Also, regarding geographic regions, a relevant north–south gradient in the frequency of pollen-food syndrome was seen with the greatest frequency reported in Northern regions in comparison to Southern Italy [26].

The presence of allergy to a given allergen source, such as birch or grass pollen, is associated with a different rate of sensitization to profilins [26,27,28,29,30]. In the already mentioned large Italian study, among patients with pollen-food syndrome, sensitization to Phl p 12 was significantly more prevalent in children allergic to melon and watermelon [26]. Allergy to different airborne allergens is also associated with different prevalence of sensitization to profilins [29,30]. Among patients sensitized to a date palm tree or a goosefoot pollen, IgE immune response to profilins (Pho d 2 and Che d 2, respectively) is detected in more than 50% of patients which classifies these proteins as major allergens [29,30]. Association between sensitization to mugwort pollen and profilins has already been reported in adult patients in northern Europe [31]. In fact, we could also demonstrate more frequent sensitization to mugwort pollen in P+ patients (not shown). Moreover, the greatest intensity of IgE ELISA to rArt v 4.0101 seen in more than 30% of P+ patients suggests that in some patients, the mugwort profilin may be the sensitizing one. In fact, this was supported by inhibition ELISA which demonstrated the greatest spectrum of Art v 4 epitopes recognized by IgE from those patients.

In a majority of patients sensitized to profilins, the greatest intensity of IgE binding was demonstrated for rPhl p 12.0101. It has been proposed that sensitization to profilins depends on the intensity of exposure to individual allergen sources, and grass pollen is the one most important [21,32]. In some populations of birch and grass allergic patients, the frequency of sensitization to profilins in those allergic to birch was greater than to grass pollen [27,28]. Interestingly, in those populations from central Europe, sensitization to profilins was more frequently seen in children than in adults [27,28]. Another study demonstrated that in a cohort of birch pollen allergic children and adults, monosensitization to birch pollen, which is frequently associated with pollen-food syndrome, was not associated with greater frequency of sensitization to Bet v 2 [33]. This observation is consistent with our results which indicate no association between sensitization to birch pollen and Bet v 2 in a group of young children and adults with pollen-food syndrome. Moreover, sensitizations to profilins were reported to be associated with high or prolonged exposure to a given allergen source and often correlated with increased severity of the disease [34,35].

Furthermore, there appears to be a correlation between age and profilin sensitization. For example, a longitudinal study of molecular spreading of sensitization to individual allergens in timothy grass allergic children demonstrated that IgE to profilins appears late and usually after sensitization to major allergens such as Phl p 1 and Phl p 5 is well developed [34]. The greatest frequency of sensitization to profilins was not achieved earlier than five years after the onset of clinical symptoms [34]. Similar observations concerning age and sensitization to profilins was made in a large population of Italian children with allergic rhinoconjunctivitis [22]. The sensitization rate to profilins was yet on the rise in allergic adolescents older than 15 years of age [22]. Moreover, clear association was demonstrated between sensitization to profilins and to the number of allergen sources including major tree, grass, and weed pollens [22]. However, in some populations of pollinosis patients older than eight years of age, no simple correlation between age and sensitization to Bet v 2 could be demonstrated [35]. We could not find any association between clinical symptoms of pollen-allergy syndrome and sensitization to profilins (data not shown). This indicates that sensitization to profilins was not necessary for development of pollen-food syndrome in young children. Moreover, it indicates that the syndrome should be dependent on sensitization to other cross-reacting allergen components such as PR-10 allergens.

Interestingly, in some populations from southern Europe, higher prevalence of sensitization to profilins in patients with different forms of plant-derived food allergy, most frequently pollen-food syndrome, was demonstrated [36]. However, in central and northern Europe, sensitization to profilins did not correlate with food allergy in allergic adults [37,38]. Moreover, in southern Europe, sensitization to Bet v 2 was not associated with pollen-food syndrome among birch allergic patients [33]. Furthermore, in those sensitized to Bet v 2, pollen-food syndrome was seen less frequently than in patients sensitized to Bet v 1 [33]. Our results are consistent with those observations. However, it is of vital importance to note that exposure to food allergenic sources may participate in immune recognition of profilins. Profilins from both wheat (Tri a 12) and soy (Gly m 3) have been described, and IgE immune response to Tri a 12 and Gly m 3 have been documented in some populations of allergic patients [39,40,41,42]. Sensitization to Gly m 3 was demonstrated in nearly 70% of patients allergic to soy products [42]. Further studies including ELISA inhibition assays with application of different pollen and food profilins may help to reveal a possible role of plant profilins in induction of IgE response in patients sensitized to those allergens.

## 4. Materials and Methods

### 4.1. Production of Recombinant Profilins

Recombinant rAmb a 8.0101, rArt v 4.0101, and rBet v 2.0101 were produced as described previously [2]. The gene coding for rPhl p 12.0101 was synthesized and inserted in pET26b plasmid and purchased from Bio Basic (Amherst, NY, USA). For easy purification by immobilized metal affinity chromatography (IMAC) using Ni-NTA, the Phl p 12.0101 construct was designed with a cleavable N-terminal 6X-polyhistidine tag, MHHHHHHSSGVDLGTENLYFQ!SGSG, where the exclamation mark shows the Tobacco Etch Virus (TEV) protease cleavage site. The newly synthesized plasmid was transformed into *E. coli* BL21(DE3) cells. A single colony was inoculated into 5.0 mL Lysogeny Broth starter culture supplemented with 100 mg of glucose and 10 µL of kanamycin at 50 mg/mL and grown with shaking (250 rpm) overnight at 37 °C. One liter cultures were then grown in terrific broth with 50 µg/mL kanamycin at 37 °C to an OD_600_ of 0.4. The cultures were cooled to room temperature (22 °C) and grown to an OD_600_ of 0.8–1.0, then induced with 0.5 mM isopropyl β-D-1-thiogalactopyranoside (IPTG), cooled to 16 °C, and grown overnight for protein expression.

After resuspending the cell pellets obtained from the 1.0 L cultures in lysis buffer (50 mM Tris-HCl, 500 mM NaCl, 2% glycerol, 20 mM β-mercaptoethanol (β-ME), 10 mM imidazole, pH 7.4), the cells were lysed by sonication. The sonicated mixture was centrifuged at 9000× *g* for 10 min at 4 °C. The supernatant containing Phl p 12.0101 was purified by IMAC using Ni-NTA. The protein was eluted using elution buffer (50 mM Tris-HCl, 50 mM NaCl, 2% glycerol, 20 mM β-ME, 250 mM imidazole, pH 7.4). SDS-PAGE was used to determine the elution fractions containing Phl p12.0101. Elution fractions containing protein were pooled and dialyzed overnight at 4 °C using Pierce SnakeSkin Dialysis Tubing with a molecular weight cut-off of 3000 Da in dialysis buffer (50 mM Tris-HCl, 150 mM NaCl, pH 7.4). The dialyzed protein was concentrated using an Amicon Ultra centrifugal filter with 3000 Da molecular weight cut-off and further purified by size exclusion chromatography in the same buffer used in dialysis. Based on size exclusion chromatography, rPhl p 12.0101 was eluted in monomeric form. TEV protease was used to cleave the purification polyhistidine-tag when needed. After concentrating the protein, the concentration of rPhl p 12.0101 was determined at 280 nm using a Thermo-Scientific Nanodrop (extinction co-efficient of 18,450 L mol^−1^ cm^−1^, as determined by ProtParam; https://web.expasy.org/protparam/). The yield of rPhl p 12.0101 was 70 mg/L culture. To cleave the purification tag from the purified protein, TEV protease was used according to the protocol described previously [43].

### 4.2. Differential Scanning Fluorimetry

Differential scanning fluorimetry (DSF) is a very robust method to study the thermal stability of proteins. Profilins, as such, have been denoted as thermally labile [10]. To investigate the thermal stability of Phl p 12 with and without the purification tag, DSF was performed by using an in-house sodium chloride salt and pH gradient screen. All the solutions prepared and the steps followed during the experiment were carried out according to a previously published protocol [43].

Briefly, 50 mM working concentration of buffer was used for all the conditions with a pH range of 4.0–9.5 (in 0.5 unit increments) and a salt range (NaCl) of 0–1.0 M (no salt, 0.125, 0.250, 0.500, 0.625, 0.750, 0.875, and 1.000 M). The DSF experiments were done using Bio-Rad CFX96 RT-PCR instrument and the melting temperatures were determined by in Bio-Rad CFX Manager software. An average melting temperature (T_m_) for the protein was found by determining the inflection point of the melting curves generated by the software (in triplicates). The melting temperatures obtained for Phl p 12 were compared to previously determined melting temperatures for Amb a 8, Art v 4, and Bet v 2 [43].

### 4.3. CD Spectroscopy

Although thousands of crystallization conditions were tested to crystallize rPhl p 12.0101, no diffracting crystal has been obtained. To estimate the secondary structure of the protein, circular dichroism (CD) spectroscopy using the JASCO spectrophotometer was employed. rPhl p 12.0101 samples for CD experiments (without purification tag) were desalted using a prepacked Sephadex^TM^ G25 column (GE Healthcare) with beads previously equilibrated in 10 mM sodium phosphate buffer that had the pH adjusted to 7.4 using phosphoric acid. The samples were concentrated to 40 μM following the same method as described above. The instrument was operated according to the manufacturer’s protocol at a wavelength range of 220–320 nm. CAPITO software was used for data processing and the secondary structure percentages were calculated [19].

### 4.4. Selection of Patients and Antibody Binding Studies

One hundred allergic patients, including 50 children and 50 adults sensitized to pollen and plant-derived food allergens, were included. Patients with parasitic infection, autoimmune and systemic diseases, chronic respiratory diseases of non-allergic background, systemic steroids, or immunosuppressive therapy were excluded from the study. The included patients had never been treated with allergen immunotherapy before the study.

The sensitization was diagnosed by the assessment of specific IgE (sIgE) antibodies to individual allergen sources using a multiparameter immunoblot polycheck (Biocheck, GmbH, Münster, Germany) according to the manufacturer’s protocol. Sensitization to individual components of birch and timothy grass pollens including Bet v 1, Bet v 2, Bet v 4, Bet v 6, Phl p 1, Phl p 5, Phl p 7, and Phl p 12 was determined using a multiparameter immunoblot (Euroline DPA-Dx Euroimmun, Lubecke, Germany) according to the manufacturer’s protocol. The detection limit of the tests is 0.35 kU/L IgE; measurable specific IgE was defined as a positive test result if >0.35 kU/L.

All sera were also tested for IgE reactivity to a panel of recombinant pollen profilins rAmb a 8.0101, rArt v 4.0101, rBet v 2.0101, and rPhl p 12.0101 which previously had been structurally and immunologically characterized [5]. The proteins were used in an enzyme-linked immunosorbent assay (ELISA) as previously described [5]. Briefly, 96-well MaxiSorp microtiter plates (Nunc, Roskilde, Denmark) were coated with 100 µL of rArt v 4.0101, rAmb a 8.0101, rPhl p 12.0101, or rBet v 2.0101 per well, each at a final concentration of 10 µg/mL diluted in coating 25 mM carbonate/bicarbonate buffer pH 9.4 and incubated overnight at 4 °C. After washing with Tris-buffered saline supplemented with 0.05% Tween 20 (TBST) buffer, the plates were blocked with TBST + 1% bovine serum albumin (BSA) for 1 h at room temperature. For IgE ELISA, the plates were washed in TBST and patients’ sera diluted in TBST + 1% BSA (1:5) were loaded on the plates and incubated for 2 h at room temperature. After the plates were thoroughly washed in TBST, a monoclonal anti-human IgE antibody conjugated to alkaline phosphatase diluted 1:1000 in TBST + 1% BSA (BD Pharmingen, Heidelberg, Germany) was applied and incubated for 2 h at room temperature. Following washing, bound antibodies were detected by incubation with p-nitrophenyl phosphate (Sigma Fast p-nitrophenyl phosphate tablet sets, Sigma-Aldrich). The reaction was quantified by measuring color intensity at 405 nm and the results presented as optical density (OD). To evaluate specificity of the test in all experiments, negative controls were used and consisted of the following: no allergen immobilized on the plate, no serum applied, irrelevant allergen immobilized on the plate, or no anti-IgE added. All samples were run in triplicate, and the mean value was used for analysis. The cut-off for positive values was determined using the mean OD values of 10 non-allergic patients’ sera plus three standard deviation (SD) values.

For the ELISA inhibition assay, in addition to regular ELISA performed as described above, patients’ sera were pre-incubated for 30 min at RT with a serial 10-fold dilutions of inhibitory allergens dissolved in TBST + 1% BSA at concentrations up to 50 μg/mL. The samples were then loaded on a plate and the procedure was continued as described for the ELISA assay above.

The study was approved be the local Bioethics Committee (R-I-002/160/2016 and R-I-002/161/2016 for children and adults, respectively). The adult patients signed a written informed consent. The parents signed a written informed consent for their children to participate in the study.

### 4.5. Statistical Analysis

Statistical analysis was performed by using SPSS for Windows software (version 13.1; PL). The comparison of quantitative variables was carried out using the Student’s t-test in case of normally distributed data and the Chi-squared and Mann–Whitney test in case of nonparametric data. A *p*-value < 0.05 was considered statistically significant. For correlation analysis, Spearman correlation test was applied.

## 5. Conclusions

In summary, our study demonstrates that the presence of IgE to profilins in patients with pollen-food syndrome is quite frequent. However, no clinical significance of that sensitization among patients living in the area where birch pollen is a dominant tree allergen source could be demonstrated. Ragweed is not present in the North–East Poland, but we observed reaction to rAmb a 8.0101. This is caused by the high sequence and structure similarity between the profilin originating from ragweed and other studied profilins. Further studies are warranted to clarify the role of individual allergen sources in the process of sensitization to individual proteins among allergic patients exposed to different aeroallergens.
